# Low-Temperature-Processed High-Performance Pentacene OTFTs with Optimal Nd-Ti Oxynitride Mixture as Gate Dielectric

**DOI:** 10.3390/ma15062255

**Published:** 2022-03-18

**Authors:** Yuan-Xiao Ma, Pui-To Lai, Wing-Man Tang

**Affiliations:** 1 School of Integrated Circuits and Electronics, MIIT Key Laboratory for Low-Dimensional Quantum Structure and Devices, Beijing Institute of Technology, Beijing 100081, China; yxma@eee.hku.hk; 2 Department of Electrical and Electronic Engineering, The University of Hong Kong, Pokfulam Road, Hong Kong Island, Hong Kong 999077, China

**Keywords:** high-k dielectric, organic thin-film transistor, high carrier mobility and small threshold voltage

## Abstract

When processed at a low temperature of 200 °C, organic thin-film transistors (OTFTs) with pentacene channel adopting high-k Neodymium-Titanium oxynitride mixtures (NdTiON) with various Ti contents as gate dielectrics are fabricated. The Ti content in the NdTiON is varied by co-sputtering a Ti target at 0 W, 10 W, 20 W and 30 W, respectively, while fixing the sputtering power of an Nd target at 45 W. High-performance OTFT is obtained for the 20 W-sputtered Ti, including a small threshold voltage of −0.71 V and high carrier mobility of 1.70 cm^2^/V·s. The mobility improvement for the optimal Ti content can be attributed to smoother dielectric surface and resultant larger overlying pentacene grains as reflected by Atomic Force Microscopy measurements. Moreover, this sample with the optimal Ti content shows much higher mobility than its counterpart processed at a higher temperature of 400 °C (0.8 cm^2^/V·s) because it has a thinner gate-dielectric/gate-electrode interlayer for stronger screening on the remote phonon scattering by the gate electrode. In addition, a high dielectric constant of around 10 is obtained for the NdTiON gate dielectric that contributes to a threshold voltage smaller than 1 V for the pentacene OTFT, implying the high potential of the Nd-Ti oxynitride in future high-performance organic devices.

## 1. Introduction

Since the discovery of semiconducting organic materials, much effort has been made to extensively explore organic thin-film transistors (OTFTs) due to their promising advantages of large–area application, low cost, flexibility and compatibility with the Si-based technology [1,2]. Thanks to continual development, the key parameter, the carrier mobility of OTFTs, has been improved to over 10 cm^2^/V·s for single-crystal organics [3] and over 2 cm^2^/V·s for amorphous thin films [4]. OTFTs have demonstrated electrical characteristics comparable to those of amorphous-silicon TFTs, and as a result, are integrated on a chip in novel applications [5]. One important application of OTFTs is to drive organic-light-emitting-diodes (OLEDs), which promotes flexible flat-panel displays. Another application is for various sensors to monitor gas, pressure, and deformation. Additionally, OTFTs have also served applications including radio-frequency identification (RFID) tap, electronic skin, and flexible circuits [6]. Although some OTFTs have demonstrated good performance, their threshold voltages are usually larger than 3 V, thus limiting their applications in portable electronic products powered by a battery at a few volts [7]. One way to lower the operating voltage of OTFTs with the traditional Si dioxide as the gate dielectric is thinning the dielectric, but at the expense of increased gate leakage. Other efforts, such as using organic-inorganic dielectrics and cross-linking passivation agents, have also been made to reduce power consumption while complicating the fabrication process [8]. As a result, attention has been paid to high-k materials adopted as gate dielectrics in field-effect transistors (FET). One famous example is Intel’s 45-nm technology node, where high-k hafnium oxide (HfO_2_) was used as the gate dielectric for Si-based metal-oxide-semiconductor field-effect transistors (MOSFETs) [9]. On the other hand, pentacene OTFT with HfO_2_ gate dielectric demonstrated a carrier mobility lower than 0.1 cm^2^/V·s because HfO_2_ had oxide defects such as oxygen vacancies and dangling bonds, producing a low-quality interface with the overlying organic channel [10]. Furthermore, other high-k materials, for example, pure tantalum oxide (Ta_2_O_5_) and titanium oxide (TiO_2_), have also been utilized as gate dielectrics for threshold-voltage reduction, but the carrier mobility was around 1 cm^2^/V·s [11].

As a lanthanide oxide, neodymium oxide (Nd_2_O_3_) has shown a high potential to act as a high-k gate dielectric for FETs. Nd_2_O_3_ has a dielectric constant of around 20, which is much higher than that of the traditional silicon dioxide (SiO_2_) (3.9), but severely suffers from high hygroscopicity, meaning stability problem when exposed to air [12,13]. With a high k value up to 80 for Ti oxide, titanium (Ti) doping in gate dielectric has been explored to raise its dielectric constant and moisture resistance [14]. However, Ti oxide is always associated with a high trap density and a smaller bandgap (~3.2 eV) than Nd_2_O_3_ (~5 eV), both increasing oxide leakage [15,16]. Therefore, it can be expected that for an Nd-Ti oxide mixture, Nd could passivate the oxide defects to suppress the leakage problem induced by Ti, while Ti could reduce the hygroscopic concern caused by Nd. As a result, high-performance OTFTs with high carrier mobility and a low threshold voltage can be obtained by using the mixture as a gate dielectric. This work aims to exploit Nd-Ti oxynitrides with various contents as gate dielectrics for pentacene OTFTs, with the goal of obtaining high device performances in terms of high carrier mobility and small threshold voltage.

## 2. Materials and Methods

**Figure 1:**
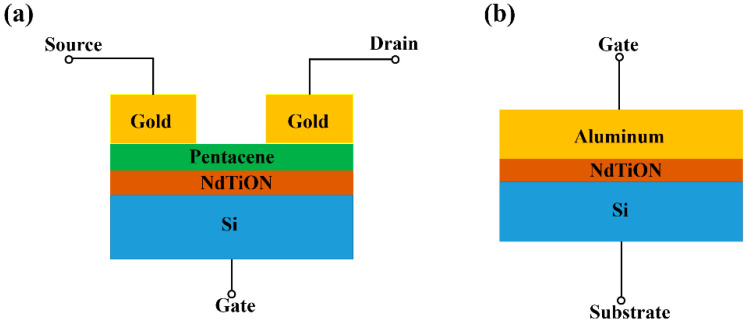
Device cross-section: (**a**) OTFT, and (**b**) capacitor.

Initially, silicon wafers (n-type, <100>, 0.005 Ω·cm) were cleaned by the standard RCA (Radio Corporation of America) method: (step 1) dipped in 5% hydrofluoric acid for 15 s, (step 2) boiled in deionized water:NH_4_OH:H_2_O_2_ (5:1:1) at 80 °C for 10 min, and (step 3) boiled in deionized water:HCl:H_2_O_2_ (5:1:1) at 80 °C for 10 min. Then, the wafers were dipped in 5% HF acid again for removal of native oxide. As a gate dielectric, pure Nd oxynitride and Nd-Ti oxynitride films with thicknesses of around 50 nm were then deposited by a sputterer (Denton Vacuum LLC Discovery 635, Moorestown, NJ, USA) on the wafers in an ambient of 30/6/3-sccm Ar/O_2_/N_2_. During the sputtering, the DC/RF powers for the Ti/Nd targets were set as 0 W/45 W, 10 W/45 W, 20 W/45 W and 30 W/45 W, respectively, denoted as samples A, B, C and D. Next step was annealing all the samples at 200 °C in N_2_ for 20 min. This was followed by deposition of 45-nm pentacene (99% purity, purchased from Luminescence Technology Corp, New Taipei City, Taiwan.) on the dielectrics by a thermal evaporator (Angstrom Engineering Nexdep, Kitchener, ON, Canada), where the deposition rate and pressure were fixed at around 1.2 nm/min and 5.3 × 10^−6^ torr, respectively. Finally, 100-nm source and drain electrodes were formed by thermally evaporating gold through a shadow mask on the pentacene film, obtaining a 100-μm-width and 40-μm-length channel. To investigate the capacitance-voltage characteristics of the gate dielectrics, dummy samples for capacitors were also cleaned by the RCA method and then received the dielectric deposition simultaneously with the TFT samples. Then, the Al electrode was thermally evaporated on these dummy samples to form capacitors with an Al/dielectric/n + Si structure, where UV lithography and lift-off method were used. An HP 4284A precision LCR meter was used for capacitor measurement, and the current-voltage curves of the OTFTs were characterized by an HP 4145B semiconductor parameter analyzer. A Wvase 32 ellipsometer was used to measure the dielectric thickness. The schematics of device structures are shown in Figure 1, and all the samples were measured in the air at room temperature.

## 3. Results and Discussion

**Figure 2:**
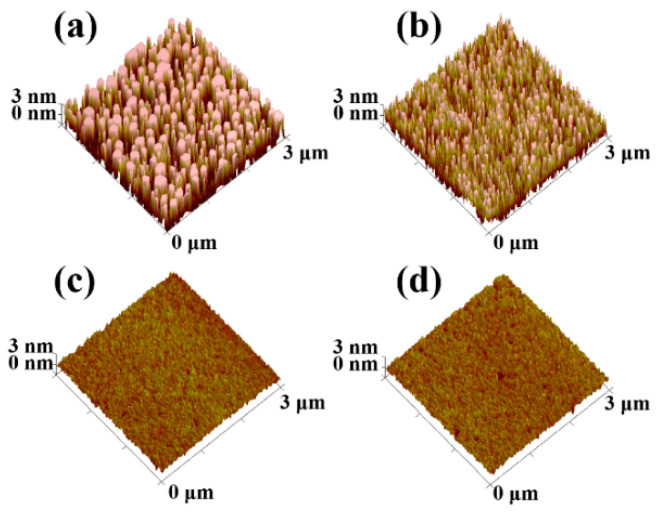
3D Atomic Force Microscopy (AFM) height-profile images of gate dielectric films with increasing Ti content: (**a**) sample A, (**b**) sample B, (**c**) sample C, and (**d**) sample D.

**Figure 3:**
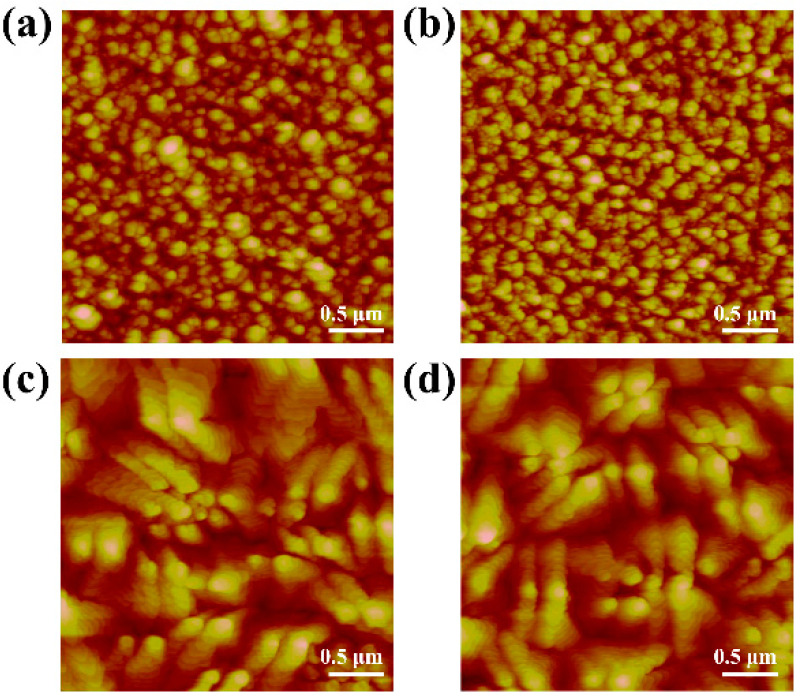
2D AFM images (3 μm × 3 μm) of pentacene films evaporated on the gate dielectrics with rising Ti content: (**a**) sample A, (**b**) sample B, (**c**) sample C, and (**d**) sample D.

Figure 2 shows the surface morphology of the gate dielectric films, which can greatly influence the performance of the OTFTs. As reflected by the root-mean-squared (RMS) values of surface roughness, the pure Nd oxynitride of sample A has the roughest surface (RMS = 7.98 nm). This rough dielectric surface should be attributed to the strong moisture absorption of Nd oxynitride as proved by X-ray photoelectron spectroscopy (XPS) for O1s spectrum in previous work [17], where the peak intensity of Nd-OH bonds was strong for pure Nd oxide but was reduced by doping the oxide with another element. Localized moisture absorption on the oxide surface causes its non-uniform expansion and thus its roughness. By fixing the sputtering power of the Nd target at 45 W and raising that of the Ti target to increase the Ti content in the dielectric, the dielectric surface roughness is obviously reduced with rising Ti content from sample A to sample D, indicating the suppressed hygroscopicity of the dielectric by the Ti incorporation, which is similar to the past works based on La oxide and Nd oxide [17,18]. The surface morphology of the pentacene films grown on the dielectrics is shown in Figure 3. Raising the Ti content in the gate dielectric, the pentacene grains become more uniform and larger due to fewer nucleation sites on smoother dielectric for pentacene growth, thus increasing the sizes of the overlying pentacene grains [19].

**Figure 4:**
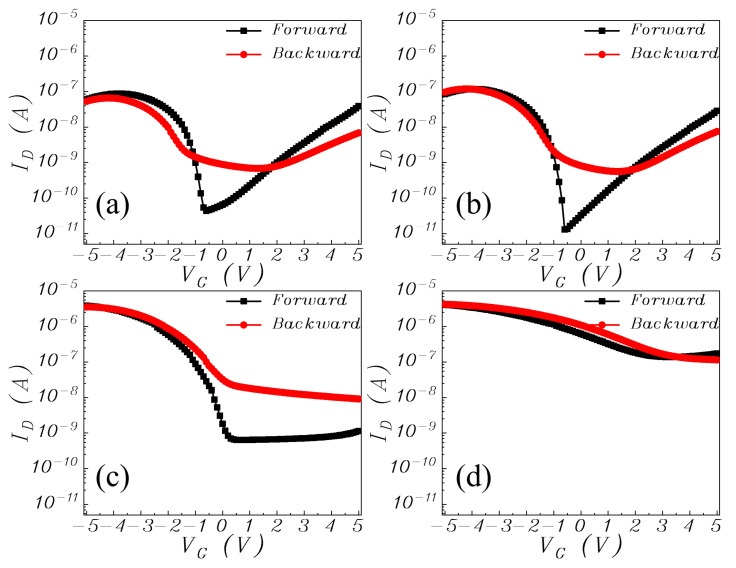
Transfer characteristics of the OTFTs with increasing Ti content in gate dielectric: (**a**) sample A, (**b**) sample B, (**c**) sample C, and (**d**) sample D.

Figure 4 displays the transfer characteristics of the samples measured at a drain-to-source voltage (V_DS_) of −5 V. The transfer hysteresis is obtained by sweeping the gate voltage from +5 V to −5 V (forward) and then reversely (backward). The difference between the threshold voltage *V_T_* of the backward sweep and that of the forward sweep is used to define hysteresis Δ*V_T_*, which is ascribed to charge trapping at/near the channel and dielectric interfaces. In Figure 4, negative Δ*V_T_* is observed for sample A, resulting from OH ions formed by the high hygroscopicity of Nd_2_O_3_ at/near the pentacene/dielectric interface [20]. These OH ions can act as donor-like traps, and during the forward sweep, capture the holes in the pentacene layer to become positively, thus producing an electric field opposite to the applied gate field [21]. Therefore, the *V_T_* negatively shifts in the ensuing backward sweep. The hysteresis is reduced for sample B, which should be due to suppressed moisture absorption of the dielectric by the Ti doping. Furthermore, the hysteresis decreases for sample B and further becomes positive for sample C and sample D, which can be ascribed to the fact that Ti doping can improve the moisture resistance of the gate dielectric and so suppress the generation of donor-like traps while inducing acceptor-like traps [16]. As previously investigated by XPS, one origin of acceptor-like traps should be the Ti-induced oxygen vacancies, which can raise the binding energy of Ti_2p_ in the XPS spectrum because they are usually considered positively-charged [22,23]. When injected from the gate electrode, electrons can be captured by these acceptor-like traps, which become negatively charged to produce an electric field parallel to the applied gate field. Therefore, the charged traps can enhance the gate-oxide field to produce a positive shift for *V_T_*.

The carrier mobility (*µ*) can be extracted from the saturation regime of p-type OTFT [24,25]:

(1) $$I_{d}=-\frac{WC_{ox}\mu }{2L}{\left(V_{G}-V_{T}\right)}^{2}$$

(2) $$\mu =\frac{2L}{WC_{ox}}{\left(\frac{\partial \sqrt{I_{d}}}{\partial V_{G}}\right)}^{2}$$

where *V_G_* is the applied gate voltage; *C_ox_* is the dielectric capacitance per unit area; *W* and *L* are the channel width and channel length, respectively.

The subthreshold swing (*SS*) and corresponding trap density (*N_t_*) can be expressed as the following formulas [26,27]:

(3) $$SS=\frac{\partial V_{G}}{\partial \left(log_{10}I_{D}\right)}$$

(4) $$N_{t}=\frac{C_{ox}}{e}\left(\frac{e\times SS}{kT\times ln10}-1\right)$$

**Figure 5:**
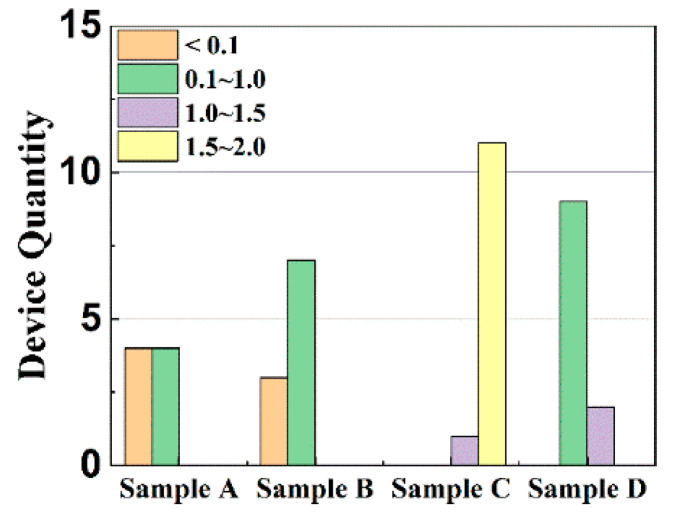
Carrier-mobility distribution for pentacene OTFTs with increasing Ti content in gate dielectric.

Extracted from their transfer characteristics, the key parameters of the samples are listed in Table 1, which are based on the averages of around 10 devices for each sample. The mobility is firstly improved with rising Ti content in the gate dielectric from sample A to sample C. This improvement should be due to the suppressed dielectric hygroscopicity and thus reduced dielectric surface roughness induced by the Ti doping (see Figure 2), which weakens the surface-roughness scattering [28,29]. Moreover, carriers can also be scattered when moving across pentacene grain boundaries, named grain-boundary scattering [24,30]. With a smoother dielectric surface, the size of overlying pentacene grains increases obviously, as depicted in Figure 3, contributing to less grain-boundary scattering from sample A to sample C. Owing to the suppressed scatterings from both surface roughness and grain boundary, sample C has the highest mobility although its *SS* and *N_t_* are slightly higher. However, the mobility is then reduced for sample D with excessive Ti content. As reflected by the trap density calculated from the extracted subthreshold swing in Table 1, excessive Ti can induce more undesirable oxide defects (possibly including oxygen vacancies and dangling bonds) to act as traps and ions at/near the interface between pentacene and gate dielectric, generating severe Coulomb scattering [31]. In summary, the carrier mobility can be degraded by larger dielectric roughness and more oxide defects generated from excessive Nd and Ti contents, respectively. Therefore, the highest mobility is achieved for sample C with appropriate Ti content. Figure 5 shows the carrier-mobility distribution where bar color and bar height represent mobility range and device quantity, respectively, thus statistically supporting the mobility trend with Ti content.

**Figure 6:**
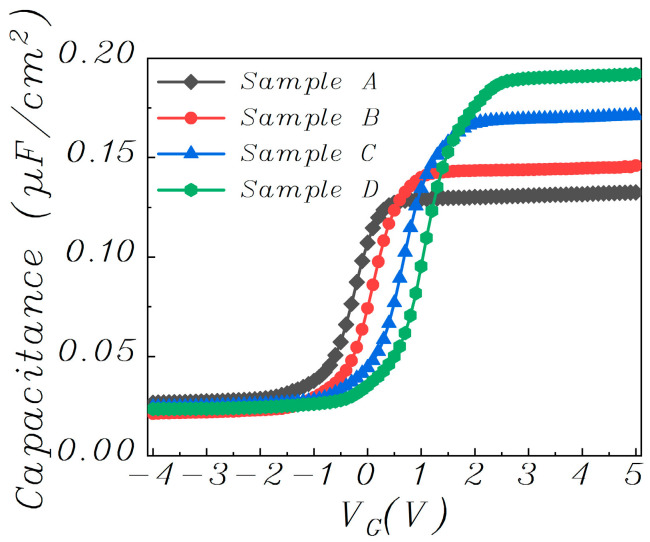
Capacitance-voltage characteristics of the capacitors with increasing Ti content in dielectric from Sample A to Sample D.

**Figure 7:**
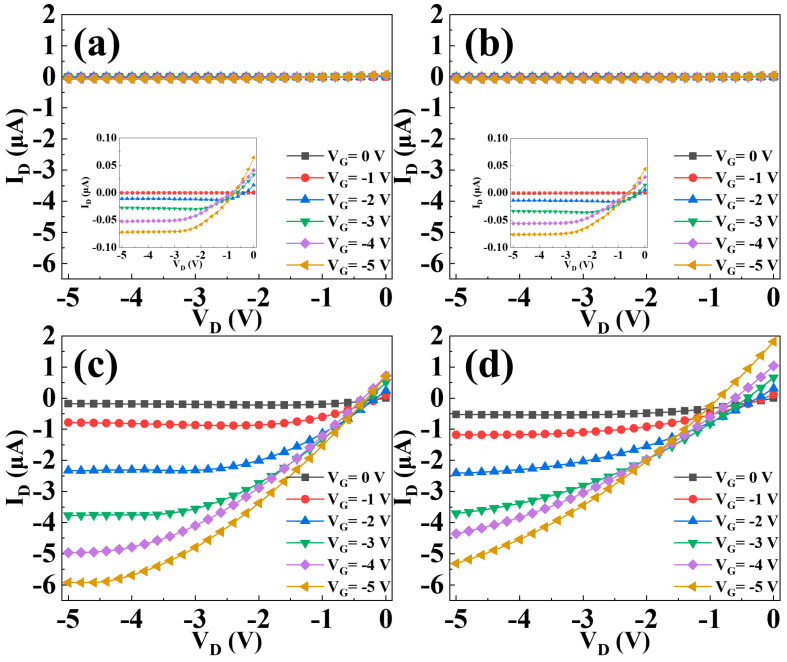
Output characteristics of the OTFTs with increasing Ti content in gate dielectric: (**a**) sample A, (**b**) sample B, (**c**) sample C, and (**d**) sample D.

**Figure 8:**
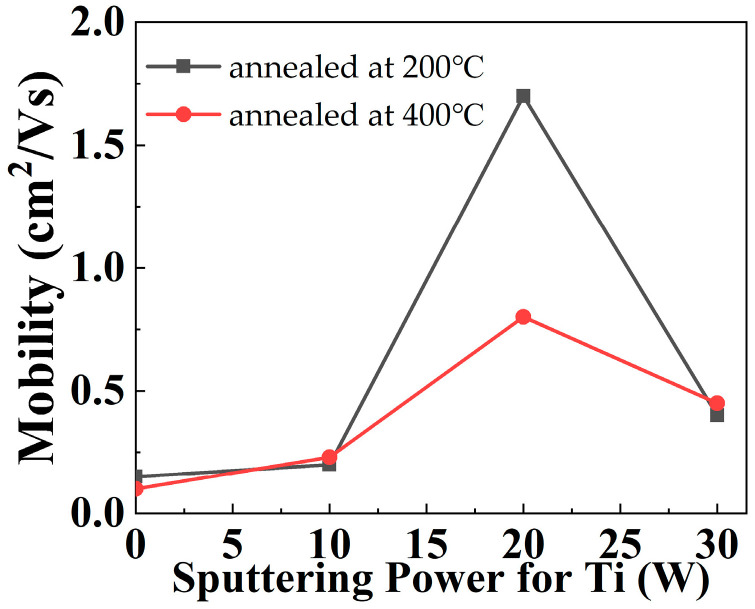
Carrier-mobility comparison for OTFTs with gate dielectrics annealed at two temperatures.

As shown in Figure 6, the capacitance-voltage (C-V) characteristics are obtained by sweeping the gate voltages of the capacitors with a structure of Al/dielectric/n + Si from ‒4 V to 5 V. The C-V curve shifts positively from sample A to sample D, which corresponds to a transition of dominance from donor-like trap to acceptor-like trap in the gate dielectric due to its increasing Ti content. Moreover, the *C_ox_* and resultant k values of the gate dielectric rise with increasing Ti content due to the higher k value of TiO than NdO. As a result, the magnitude of the threshold voltage is reduced from sample A to sample C. Strangely, sample D shows a positive threshold voltage for p-type pentacene channel, which should be the result of more negative charges induced by excessive Ti, as supported by the dielectric–charge density extracted from the CV characteristics listed in Table 1. The negative oxide charges can turn on the device even without an externally applied voltage. The leakage current of the gate dielectric can be reflected by the drain current (under V_D_ = V_S_ = 0 V) in the output characteristics shown in Figure 7. The largest gate-dielectric leakage of sample D should originate from the highest Ti content in its dielectric because (1) Ti incorporation can generate traps in the dielectric as supported by the highest *N_t_* of sample D in Table 1; and (2) Ti oxide has a smaller band gap (~3.4 eV) than Nd oxide (~5.0 eV). In [32], the Nd-Ti oxynitrides were deposited simultaneously with those in this work, but were annealed at a higher temperature of 400 °C. The pentacene OTFTs with these Nd-Ti oxynitrides as gate dielectrics also showed the highest mobility for the sample with Ti sputtered at 20 W (see Figure 8). Interestingly, though higher annealing temperature normally improves the quality of the gate-dielectric/semiconductor interface to result in higher mobility, NdTiON annealed at 400 °C gives the OTFT lower carrier mobility (0.8 cm^2^/V·s) than that annealed at 200 °C (1.7 cm^2^/V·s). This should originate from the chemical reaction at the remote Si-gate/gate-dielectric interface, which is affected by the annealing temperature. Higher annealing temperature increases the chemical reaction rate to produce a thicker interlayer, which reduces the gate screening effect on the remote phonon scattering to degrade the carrier mobility, which has been investigated for various lanthanide-based high-k gate dielectrics (including LaTiON and NdTaON) for OTFTs [33,34].

## 4. Conclusions

Using Nd-Ti oxynitrides with different Ti contents as gate dielectrics, pentacene OTFTs have been fabricated at a low temperature of 200 °C. The oxynitrides are obtained by co-sputtering Ti and Nd targets at variable DC power and fixed RF power, respectively. The surface roughness of the NdTiON film is reduced with increasing Ti content to produce larger overlying pentacene grains, which should be due to the reduced hygroscopicity of the gate dielectric. The improved qualities of NdTiON and pentacene contribute to the smallest threshold voltage (−0.71 V), and the highest carrier mobility (1.70 cm^2^/V·s) for the sample with the Ti target sputtered at 20 W for the gate dielectric. This sample also shows much higher mobility than its counterpart processed at a higher temperature of 400 °C (0.8 cm^2^/V·s) because its thinner gate-dielectric/gate-electrode interlayer can provide stronger screening on the remote phonon scattering by the gate electrode. However, numerous acceptor-like traps are induced by excessive Ti sputtered at 30 W, thus reducing the carrier mobility and increasing the *V_T_* hysteresis. Moreover, owing to the high-k value of the gate dielectric, the threshold voltage of the sample can be smaller than 0.8 V, implying its high potential to lower the power consumption of future OTFTs.

## Figures and Tables

**Table 1:** Main electrical parameters of OTFTs with increasing Ti content in gate dielectric.

Sample	A	B	C	D
RF Sputtering Power of Nd	45 W			
DC Sputtering Power of Ti	0 W	10 W	20 W	30 W
*μ* (cm^2^/V·s)	0.15	0.20	1.70	0.40
*V_T_* (V)	−0.81	−0.75	−0.71	2.1
hysteresis (V)	−0.36	−0.23	0.33	1.0
*SS* (V/dec)	0.25	0.16	0.30	3.1
*N_t_* (cm^−2^eV^−1^)	2.6 × 10^12^	1.5 × 10^12^	4.1 × 10^12^	6.1 × 10^13^
on/off ratio	2.0 × 10^3^	1.0 × 10^4^	6.5 × 10^3^	3.7 × 10^1^
*V_fb_* (V)	−0.65	−0.28	0.19	0.58
*Q_ox_*	2.7 × 10^11^	−3.7 × 10^10^	−5.5 × 10^11^	−1.1 × 10^12^
*C_ox_* (μF/cm^2^)	0.132	0.146	0.171	0.191
*k* value	7.7	8.6	9.7	10.2
*t_ox_* (nm)	51.4	52.1	50.3	47.5
RMS dielectric roughness (nm)	7.98	2.21	0.36	0.35

## Data Availability

Data available in a publicly accessible repository.
